# Serum CD5L as potential biomarker of thyroid hormone status during pregnancy

**DOI:** 10.1002/biof.2123

**Published:** 2024-09-30

**Authors:** Sabrina Asaad, Thilo Samson Chillon, Dorota Filipowicz, Britta Wilms, Frank Strenge, Ewelina Szczepanek‐Parulska, Waldemar B. Minich, Sebastian M. Meyhöfer, Jens U. Marquardt, Jens Mittag, Henrik Oster, Marek Ruchala, Lutz Schomburg

**Affiliations:** ^1^ The Institute for Experimental Endocrinology Charité Medical School Berlin Germany; ^2^ Department of Endocrinology, Metabolism and Internal Medicine Poznan University of Medical Sciences Poznan Poland; ^3^ Institute for Endocrinology and Diabetes, Center of Brain Behavior & Metabolism University of Lübeck/Universitätsklinikum Schleswig‐Holstein Lübeck Germany; ^4^ Department of Medicine I University of Lübeck Lübeck Germany; ^5^ German Center for Diabetes Research (DZD) München‐Neuherberg Germany; ^6^ Lübeck Institute of Neurobiology University of Lübeck Lübeck Germany

**Keywords:** circadian rhythm, cirrhosis, copper, diagnostics, feedback, hypothyroidism, inflammation, selenium

## Abstract

The thyroid hormone (TH) status is routinely assessed by thyrotropin (TSH) and thyroxine (T4). Both biomarkers are mainly regulated by TH receptor beta, whereas many peripheral organs employ the alpha receptor. Serum cluster of differentiation 5‐like molecule (CD5L) is a liver‐derived protein under control of both TH receptor isoforms. However, clinical data on its relation to TH status are sparse. An additional biomarker of TH status is needed in particular during pregnancy, where the routine biomarkers become dynamically disturbed. This study aimed to determine possible covariates regulating serum CD5L and to test its potential suitability as additional TH biomarker during pregnancy. A sandwich ELISA for serum CD5L was established using newly raised antibodies. Circadian effects and the impact of liver disease on serum CD5L concentrations were assessed. Serum samples from pregnant women with well‐characterized TH and trace element status were analyzed, and CD5L concentrations were correlated with other indicators of TH status including TSH, fT4, fT3, copper, and selenium concentrations. The new quantitative assay for CD5L showed high accuracy. Serum CD5L was stable in dilution and refreezing experiments and did not show strong circadian variance or dependency on liver disease. In serum of pregnant women, CD5L correlated positively to fT3, but not to fT4 or TSH. Significant positive correlations of CD5L were observed with serum levels of the TH‐responsive trace elements selenium and copper. The data support the potential suitability of serum CD5L as an additional marker of TH status, with potential value for pregnancy and thyroid disease.

## Graphical abstract

The thyroid hormone status is routinely assessed by thyrotropin and thyroxine, but additional biomarkers are needed, in particular in pregnancy. This study describes that serum cluster of differentiation 5‐like molecule correlates in pregnant women to the active thyroid hormone fT3, and qualifies as a promising candidate to close this diagnostic gap.

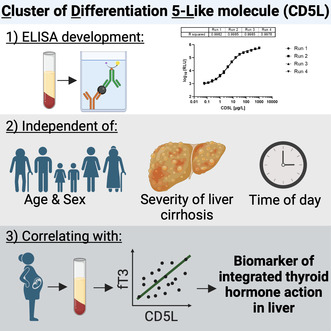

**Abbreviations**
CD5L: cluster of differentiation 5‐like molecule
CP: ceruloplasmin
Cu: copper
CV: coefficient of variation
ELISA: enzyme‐linked immunosorbent assay
FGF‐21: fibroblast growth factor‐21
fT3: free T3
fT4: free T4
IgM: immunoglobulinM
LLOQ: lower limit of quantification
mAbs: monoclonal antibodies
MELD: model for end‐stage liver disease
RLU: relative light units
SD: standard deviation
Se: selenium
SELENOP: selenoprotein P
SHBG: sex‐hormonebinding globulin
T3: tri‐iodothyronine
T4: tetra‐iodothyronine
T4: thyroxine
TH: thyroidhormone
THRA: TH receptoralpha
THRB: TH receptorbeta
TR: TH receptor
TSH: thyrotropin
ULOQ: upper limit of quantification

## 1 INTRODUCTION

The thyroid hormone (TH) axis serves as a paradigm for negative feedback regulation in the endocrine system. Thyroid gland activity is controlled by thyrotropin (TSH) from the pituitary, while TSH biosynthesis and secretion are stimulated by hypothalamic TSH‐releasing hormone (TRH). These two stimulating hormones are subject to negative feedback regulation by the major THs from the thyroid gland, that is, thyroxine (T4) and tri‐iodothyronine (3,3′,5‐T3).^1^ Both TRH and T3 display minimal blood concentrations only and are therefore not routinely quantified. Accordingly, the individual TH status of a given subject is usually analyzed by TSH concentrations, sometimes in combination with the measurement of free T4 (fT4), providing a reliable assessment in the majority of conditions. However, serum TSH shows considerable circadian variation.^2^ Moreover, some notable conditions with ambiguous TSH and fT4 results have been identified and highlight that additional markers of TH status are needed to improve assessment of the TH axis activity during clinical care.^3^, ^4^

In addition, there are rare genetic disorders affecting TH transport and metabolism, where the TH activity in a peripheral tissue seems not to be reliably reflected in the TSH/fT4 serum levels. This has been shown for genetic mutations in TH receptor alpha (THRA),^5^ in central factors controlling selenoprotein biosynthesis,^6^ or in TH transporters such as the monocarboxylate transporter.^7^ For this reason, organ‐, or TH receptor (TR)‐specific biomarkers are sought for, to enable a more accurate, and systemic view on the whole body TH status, with particular relevance for patients under treatment for hypo‐ or hyperthyroidism and for pregnant women.^4^

Finding a reliable biomarker for TH action during pregnancy is of particular importance both for the mother and the growing child, as complications during pregnancy and delivery as well as neurodevelopmental issues have been shown in hypo‐ and hyperthyroidism.^8^, ^9^ Part of the challenge is due to placenta‐derived human chorionic gonadotropin which increases steeply and causes activation of the TSH‐receptor, rendering TSH measurements for assessing TH status unsuitable.^8^ At the same time, total plasma volume changes during the course of pregnancy, affecting the concentration of biomarkers, and sex steroids impact on gene expression and concentrations of circulating TH‐binding proteins, disturbing the analytical procedures.^10^ The assessment of TH status becomes even more challenging in women with inherited or acquired thyroid dysfunction, due to autoimmune disease, autonomy, nutritional deficits, or other causes, further affecting the routine biomarkers of TH status dynamically and necessitating stage, disease‐type, and population‐specific reference intervals.^8^, ^9^, ^11^

A number of circulating proteins and metabolites have been suggested over the years, but none of the candidates have been considered suitable as a routine biomarker of TR or tissue‐specific TH activity, yet.^12^ In our previous studies, we identified five serum parameters as hepatic biomarkers of TH status, namely total selenium (Se) and selenoprotein P (SELENOP),^13^ total copper (Cu) and ceruloplasmin (CP),^14^ along with cluster of differentiation 5‐like molecule (CD5L) concentrations.^15^ Notably, serum CD5L concentrations correlated positively with fT3 and negatively with TSH, that is, qualifying as a positive TH‐dependent peripheral marker.^15^ In general, CD5L is known as an important cytokine within the immune system, eliciting an important role as central communication signal between lymphocytes.^16^ It has recently been used in experimental models of sepsis, and shown to successfully reduce morbidity and mortality rates after i.v. application of recombinant protein.^17^ Similarly, in experimental models of stroke and kidney injury, CD5L proved beneficial in reducing the neuronal and renal damage, respectively.^18^ A direct effect on intracellular metabolism of macronutrients has been described in the context of lipolysis by adipocytes after active endocytosis of CD5L which apparently inhibits fatty acid synthase.^19^

However, it seems likely that covariates other than TH also influence serum CD5L levels, and it is not known whether endocrine regulation of CD5L by TH takes precedence over, for example, circadian, liver disease‐related, or pregnancy‐related effects. Thus, it remains to be tested whether serum CD5L may indeed qualify as a biomarker of TH status in view of its nature as a macrophage‐derived hepatic cytokine. In order to address this issue, we developed and characterized a novel sandwich enzyme‐linked immunosorbent assay (ELISA) for human CD5L, assessed CD5L concentrations in serum samples from a circadian profile study, from patients with liver disease and women during pregnancy. Our results indicate good stability of CD5L in serum, robust expression independent of time of day, and consistent correlation to several indicators of TH status, thereby further supporting its potential suitability as biomarker of peripheral TH activity. The observed positive correlation of CD5L to fT3 in pregnancy warrants further studies with additional endpoints of TH action, such as child growth and neurodevelopment, to verify its suitability as additional TH biomarker during this most decisive and vulnerable period in life.

## 2 EXPERIMENTAL PROCEDURES, MATERIALS AND METHODS

### 2.1 Human samples and associated data

Three sets of human serum samples were analyzed for this study and assessed for serum CD5L concentrations. All three studies had received ethical clearance, and all human subjects enrolled had provided written informed consent prior to the analyses. The studies were performed in accordance with the principles laid down in the Declaration of Helsinki.

A group of healthy adults underwent circadian blood drawings at the University of Lübeck, Germany, providing samples from different times during the day with intervals of 2 h between the collections, as described previously.^20^ Written informed consent of the participants had been obtained prior to sampling. The ethical permission had been granted by the ethics committee of the University of Lübeck, Germany (AZ 12‐027) on February 15, 2012.

Serum samples from liver disease patients (women; *n* = 39, mean age: 57.5 ± 10.2 years, and men; *n* = 65, mean age: 58.9 ± 10.8 years) treated at University Medical Center Mainz, Germany, between 2013 and 2019 were prospectively collected. Serum samples were centrifuged at 4°C at 3000 rpm and stored within 30 min at −80°. Clinicopathological data as well as disease‐specific serum parameters have been assessed for every patient. The sampling and data processing was approved by the local ethics committee of the University Medical Center Mainz and performed in accordance with all relevant data protection criteria and the Declaration of Helsinki. All patients agreed to this study by signing an informed consent (Ethics agreement no: 837.199.10 or 837.052.12; 8153, Mainz, Germany).

A group of pregnant women were invited to participate in a clinical study conducted in the Gynecological and Obstetric Clinical Hospital at Poznan University of Medical Sciences, Poznan, Poland, as described previously.^21^ A maternal nonfasting venous blood sample was taken prior to delivery, and trace element status including serum Cu and Se concentrations were determined by total reflection x‐ray spectroscopy, as described.^21^ Parameters of TH status comprising TSH, fT4, and fT3 along with thyroid autoantibodies were determined by routine laboratory tests, as described.^21^, ^22^ All the women were recruited in the third trimester of pregnancy at admission to the obstetric ward (just before delivery, *n* = 114, mean age: 34 ± 4 years, mean body weight: 64.7 ± 11.7 kg before pregnancy, delivery at pregnancy week 38.8 ± 1.6, with 94% of deliveries at term). All the women enrolled provided written informed consent and the study had been approved by the bioethics committee of Poznan University (approved January 10, 2019, protocol no. 104/19, Poznan, Poland).

### 2.2 Establishment of a sandwich assay for CD5L

To enable a high‐volume, cost efficient, and scalable assessment of CD5L concentrations, a novel luminometric immunoassay for human CD5L (sandwich ELISA) was established. To this end, human CD5L was recombinantly expressed in insect cells, purified to homogeneity by affinity chromatography, and injected into mice yielding a set of monoclonal antibodies (mAbs), essentially as described before for other serum proteins.^23^, ^24^, ^25^ Briefly, the complete open reading frame of human CD5L with a C‐terminal His‐tag was synthesized as cDNA by a commercial supplier (Eurofins Genomics GmbH, Ebersberg, Germany) and inserted into the baculovirus transfer vector *pFastBac1* (Thermo Fisher Scientific, Karlsruhe, Germany). The resulting expression plasmid was transformed into competent *Escherichia coli* cells, and recombinant bacmid DNA was isolated. Insect Sf9 cells were transfected with bacmid DNA for obtaining a recombinant virus stock that was used to initiate CD5L protein biosynthesis in “High Five” insect suspension cells. Cell culture supernatant was harvested 48 h after infection, protein was isolated by affinity chromatography on Ni‐NTA agarose according to the manufacturer's instructions (Qiagen GmbH, Hilden, Germany) and quantified. Immunization, hybridoma generation, and mAb selection were performed in collaboration with a commercial service supplier (UNICUS Karlsburg OHG, Greifswald, Germany). A set of two mAb was selected using the recombinant protein as target structure. The hybridoma cells were cultured in high volume, the mAb were purified (ASKA Biotech GmbH, Hennigsdorf, Germany), and used to generate a CD5L‐specific luminometric sandwich detection assay (ELISA). Quality of the ELISA was tested with respect to linearity, inter‐, and intra‐assay coefficients of variation (CV), and functional assay sensitivity using recombinant protein. Stability of CD5L in serum was characterized with respect to several freeze‐thaw cycles and linear dilutions. Two formats of the ELISA were established and compared, namely a manual 96‐well format test and a laboratory automat‐based test employing the IDS‐iSYS multidiscipline automated system (ids immunodiagnostic systems, Boldon, UK).

### 2.3 Quantification of serum SELENOP and ceruloplasmin by ELISA

The concentrations of the Se‐transporter SELENOP and the cuproenzyme CP were determined by sandwich ELISA methods, using the characterized mAbs described earlier.^23^, ^24^ For this study, one antibody recognizing human SELENOP or CP was coupled to magnetic beads (dynabeads MyOne™, tosylactivated, Thermo Fisher) to serve as catcher, while the other antibody was labeled by acridiniumester‐*N*‐hydroxy‐succinimid (InVent Diagnostica GmbH, Berlin, Germany) to serve as detector of emitted signals (measured as relative light units). Assay parameters were optimized for quantification on the IDS‐iSYS laboratory automat, employing a set of standards and calibrators as described.^23^, ^24^ Results obtained by the manual and automated assays showed high concordance, and intra‐ and inter‐assay CV of both methods were below 15%.

### 2.4 Statistical analysis

The distribution of numeric variables was examined using the Shapiro–Wilk test. Non‐normal data sets are presented as median with interquartile range. Pairwise comparisons were performed using the Wilcoxon rank‐sum test to detect differences in serum markers. Spearman rank correlation was used to detect correlations between continuous variables. All statistical analyses were two‐tailed, and *p*‐values <0.05 were considered statistically significant. Statistical analyses were performed with R on RStudio (version 4.3.2).

Goodness of fit for independent duplicates of human CD5L standard calibrators was calculated using GraphPad Prism 9 software (GraphPad Software Inc., San Diego, USA). The coefficient of determination (*R* ^2^) was calculated using unweighted four parameter logistic regression.

## 3 RESULTS

### 3.1 Establishment and characterization of a sandwich ELISA for human CD5L

**FIGURE 1:**
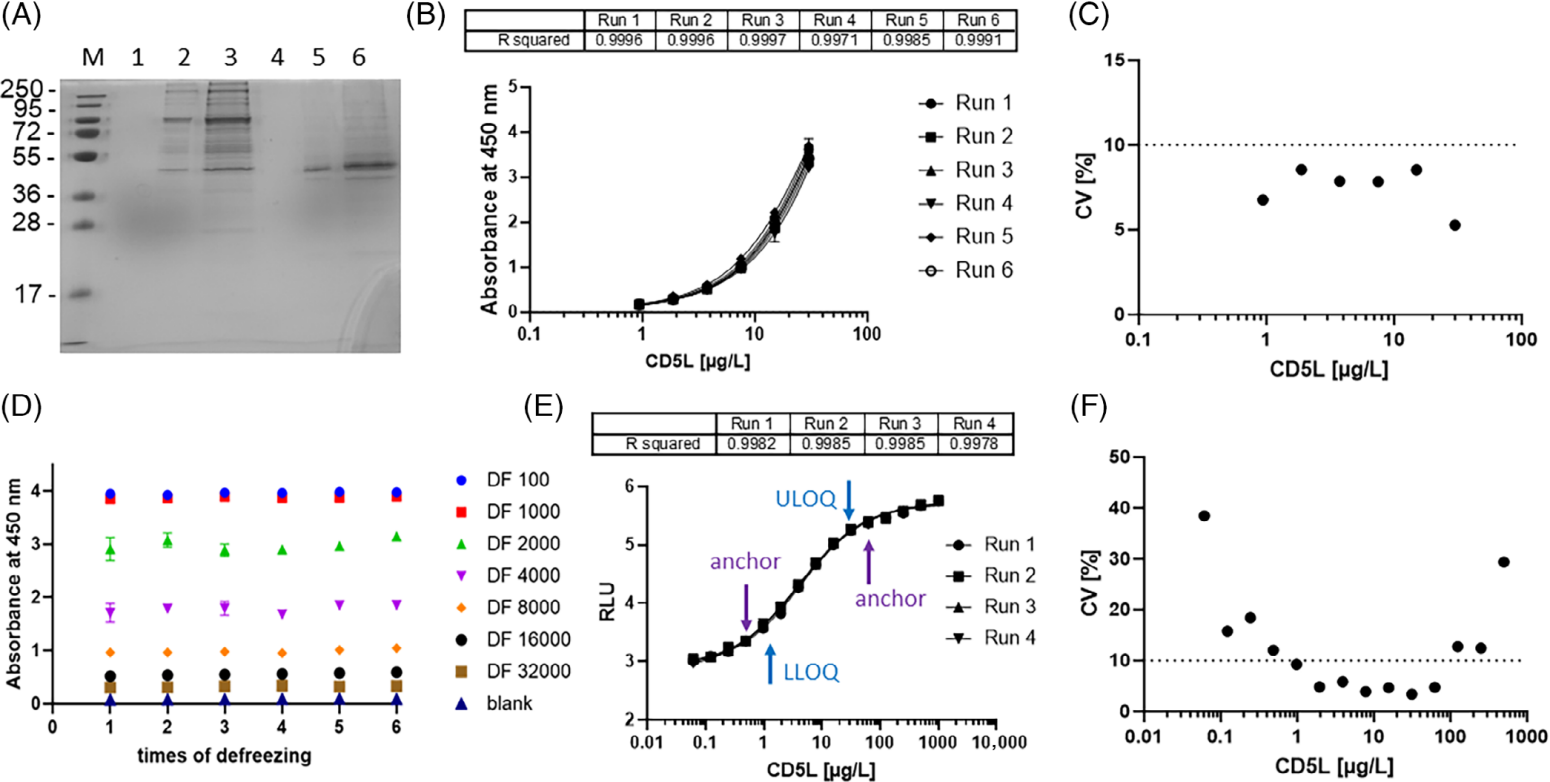
Recombinant expression of human cluster of differentiation 5‐like molecule (CD5L) and ELISA development. (A) Human CD5L was recombinantly expressed in insect cells and purified by Ni‐NTA agarose affinity. The size marker (M) is indicated, along with the stained proteins in the fractions resulting from elution using 40 mM (lanes 2, 3) or 250 mM (lanes 5, 6) imidazole. (B) After immunization, two CD5L‐mAbs were selected and used to establish a quantitative ELISA for CD5L as a manual sandwich test. Six assay runs were conducted and displayed highly reproducible dose–response curves with recombinant CD5L. (C) The CV were determined over a wide concentration range of recombinant human CD5L and indicated a relatively large functional assay sensitivity. (D) Using a standard human serum pool, the determination of CD5L proved robust to thawing, yielding comparable results from samples that underwent up to six separate rounds of defrosting. (E) Adapting the ELISA to an automated assay system yielded again highly reproducible results. (F) The functional assay sensitivity characterized by a CV of less than 10% extended over approximately two orders of magnitude. CV, coefficient of variation; M, molecular weight marker [kD]; LLOQ; lower limit of quantification; ULOQ, upper limit of quantification, anchors; points denoting slope stability in a four parameter logistic algorithm for quantitative analysis of sigmoid functions.

Human CD5L was recombinantly expressed in insect cells, purified to homogeneity and used to develop mAbs to CD5L (CD5L‐mAb; Figure 1A). Western blot analysis verified specificity and sensitivity of the antibodies, and helped to identify a pair of CD5L‐mAb suitable to generate a luminometric immunoassay in sandwich format (ELISA) for manual and automated detection. The newly established tests appeared to be robust and reliable. Six runs of the manual test were prepared and tested, yielding very congruent results in dilution experiments (Figure 1B). The inter‐assay CV was below 10% in the concentration range of 1.0–30 μg/L of CD5L (Figure 1C). A serum pool was tested for stability with respect to freezing and thawing, indicating no loss of immunogenicity upon up to six consecutive freezing cycles in different dilutions. The deviations between the CD5L signals from a given dilution were consistently below 6% across the six freeze–thaw cycles (Figure 1D). The CD5L ELISA was then adapted for automated detection, and four separate determinations were run at different time points. The results indicated reliable and reproducible quantification of CD5L (Figure 1E), yielding a quantification range extending over two orders of magnitude (Figure 1F). Higher imprecision was detected at repeated measurements of CD5L concentrations below 0.5 μg/L or above 100 μg/L.

### 3.2 Circadian variations of serum CD5L over the course of day

**FIGURE 2:**
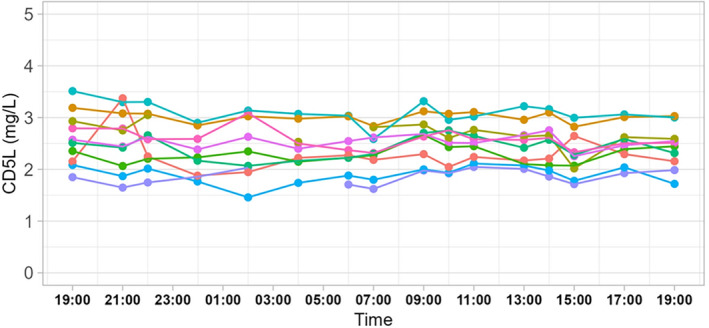
Circadian variation of serum cluster of differentiation 5‐like molecule (CD5L) concentrations in healthy subjects. Serum samples collected at different times of the day from 10 human adults were analyzed for CD5L concentrations. The results indicate some relatively stable inter‐individual differences, but no strong variations with regard to circadian rhythmicity.

A set of human samples with timed blood drawings across 24 h was analyzed by the newly generated ELISA for circadian variations of CD5L. The results indicate relatively stable signals during the day in all 10 individuals analyzed (Figure 2). Some relatively constant interindividual differences in serum CD5L concentrations were noted, in a relatively narrow concentration range of 1.9–3.1 mg/L (Table S1). The within‐subject variations ranged from 3.5% to 14.9%, with nine of the 10 subjects displaying CV‐values below 10%. These results indicate no strong effect of the time of blood drawing and little circadian variation in signal intensities with relatively constant variation in between subjects, supporting CD5L as robust and stable serum biomarker.

### 3.3 Association of serum CD5L with severity of liver disease

**FIGURE 3:**
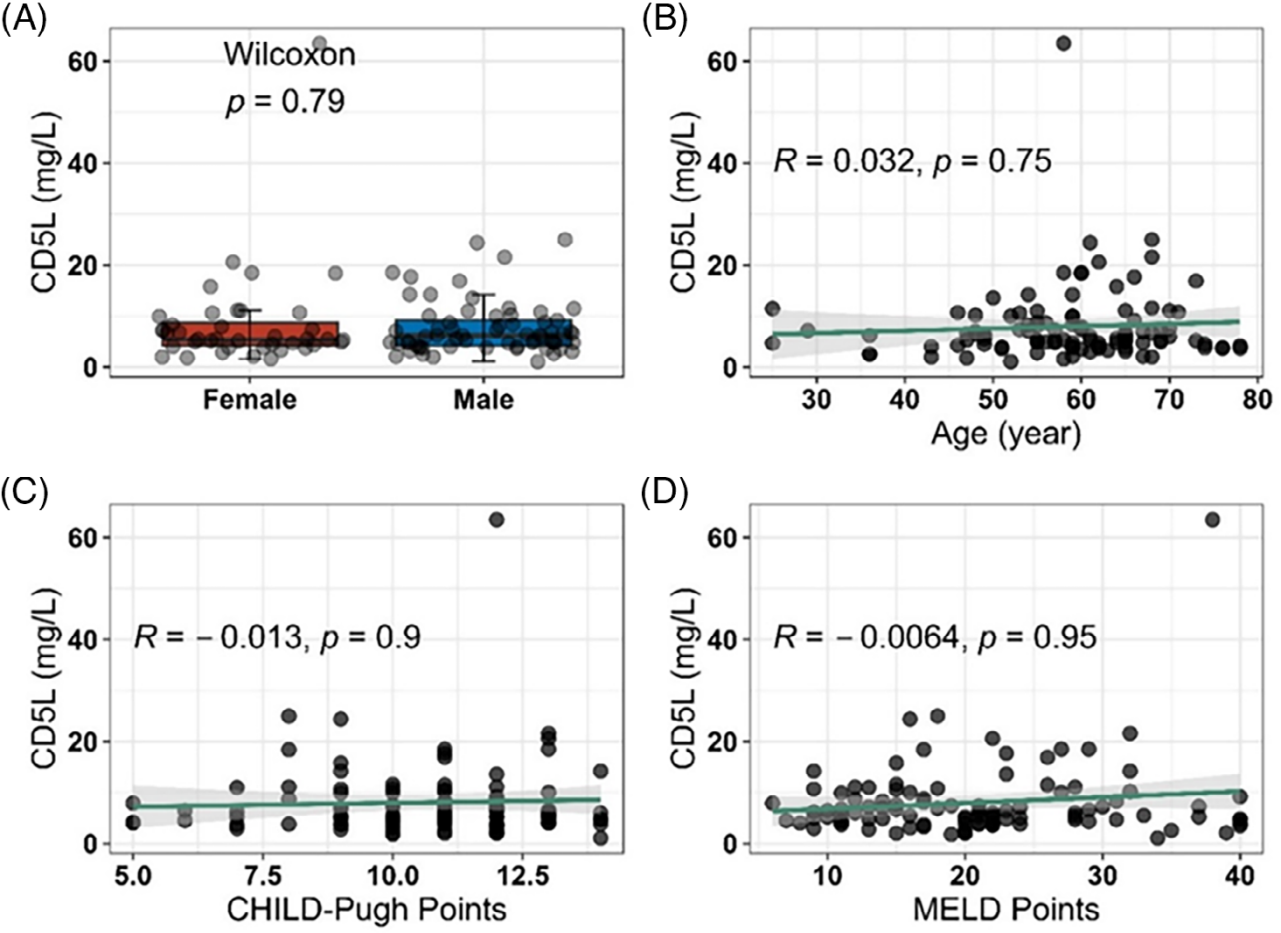
Potential variation of serum cluster of differentiation 5‐like molecule (CD5L) concentrations regarding sex, age, or stage of liver disease. Serum samples from patients with liver cirrhosis were analyzed for CD5L. Serum concentrations did not differ with regards to (A) sex, or (B) age of the patients. Severity of liver dysfunction is expressed as number of points in the CHILD‐Pugh score, and requirement for liver transplant is characterized by the number of MELD points. Serum CD5L concentrations correlated neither to (C) the CHILD‐Pugh, nor to (D) the number of MELD points. *N* = 105 samples, group comparison by nonparametric two‐sided Wilcoxon test, correlation analysis by nonparametric Spearman rank‐order correlation.

To evaluate the potential impact of an impaired liver function on hepatic CD5L release, serum samples from patients with different stages of liver cirrhosis were analyzed. A comparison of female versus male cirrhosis patients was conducted, yielding no indication for a sex‐specific difference in CD5L in this disease (Figure 3A). Similarly, no obvious dependence on age of serum CD5L concentrations was noted in the patients with liver disease (Figure 3B). Using the MELD and CHILD‐Pugh scores as indices of severity of liver dysfunction and mortality risk, respectively, no direct relationship between these liver disease parameters and serum CD5L concentration was detected (Figure 3C,D). Collectively, the data indicate no consistent impact of sex, age, or impaired liver function on serum CD5L concentrations in the patients with liver cirrhosis.

### 3.4 Correlation of serum CD5L to TH biomarkers in pregnant women before delivery

**FIGURE 4:**
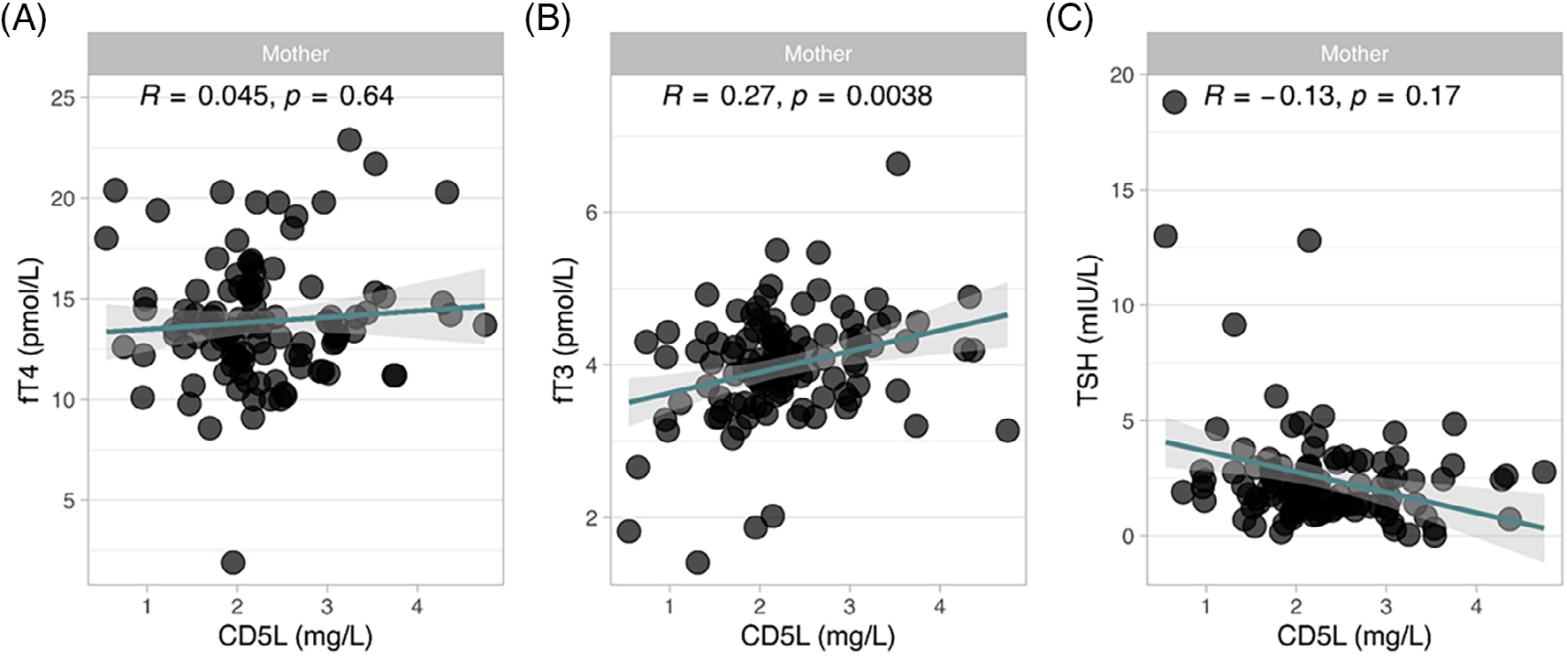
Associations of serum cluster of differentiation 5‐like molecule (CD5L) with the routine biomarkers of the thyroid hormone (TH) axis in pregnant women shortly before delivery. Serum concentration of CD5L showed (A) no significant correlation to free T4 (T4), but (B) a positive correlation to fT3. (C) No significant association between serum CD5L and thyrotropin (TSH) concentration was observed. Correlation analysis by nonparametric Spearman rank‐order correlation, *R* and *p*‐values are indicated.

Total CD5L concentrations in the samples of these pregnant women shortly before delivery showed similar concentrations as observed in the analysis of circadian samples ([CD5L], pregnancy [mean ± SD; 2.3 ± 0.8 mg/L, vs. circadian samples; 2.5 ± 0.4 mg/L]). In the samples, no significant association of serum CD5L was observed with fT4 (Figure 4A), but CD5L correlated positively with fT3 concentrations (Figure 4B). Serum CD5L and TSH showed an inverse trend, but were not significantly correlated in the samples from pregnant women (Figure 4C).

### 3.5 Comparison of CD5L to Se, Cu, SELENOP, and CP as alternative markers of TH activity

**FIGURE 5:**
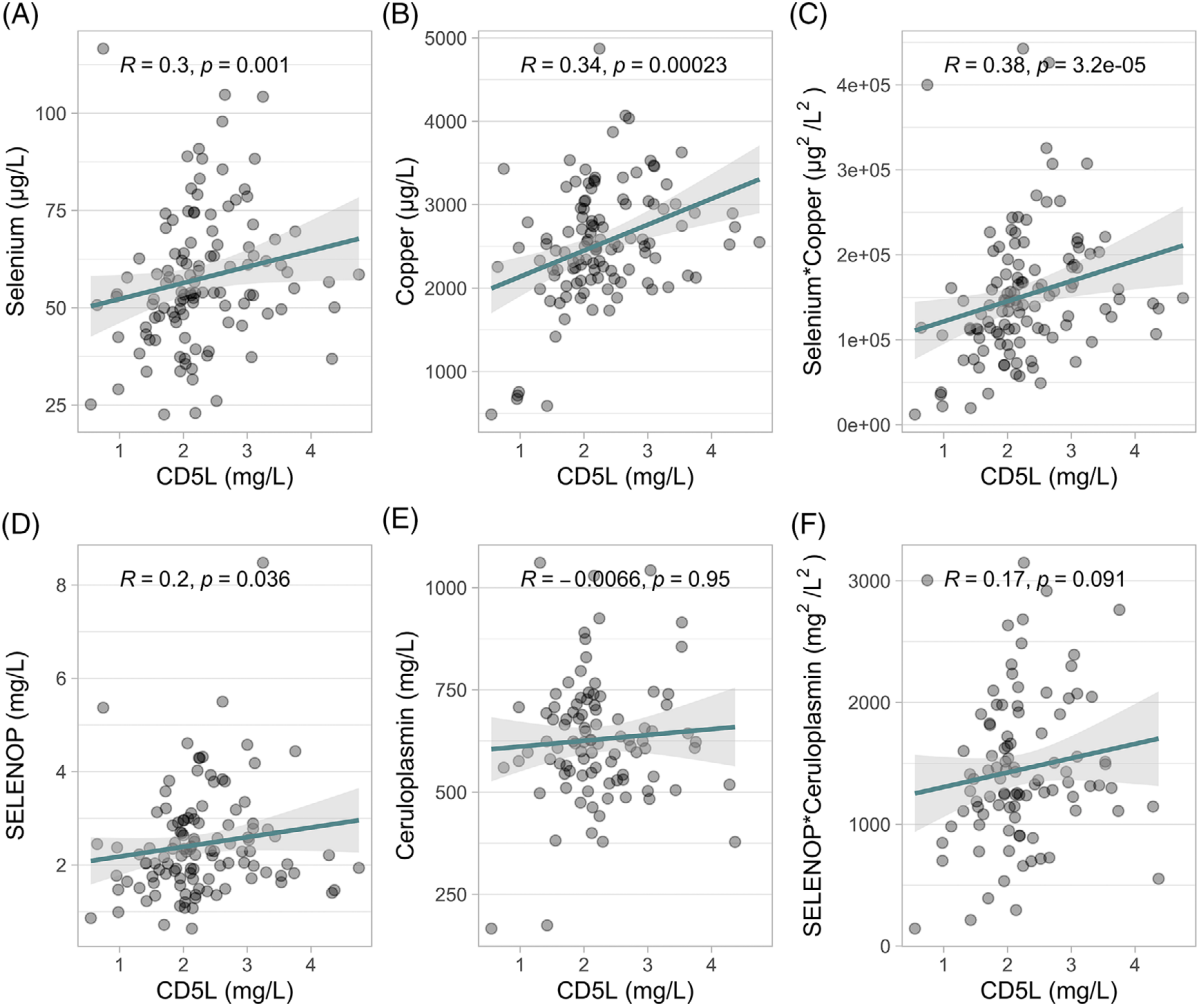
Associations of serum cluster of differentiation 5‐like molecule (CD5L) in pregnancy with serum biomarkers of Se and Cu status. The serum CD5L concentrations in pregnant women showed significant positive correlation with (A) total serum Se, (B) total serum Cu, and (C) the product of total serum Cu and total serum Se concentrations. In comparison, (D) serum CD5L concentrations were significantly associated with serum selenoprotein P (SELENOP), but not with (E) serum CP or (F) the product of serum SELENOP and CP in pregnancy. Comparisons by nonparametric rank‐order Spearmen correlation; the *R* and *p*‐values are indicated as inset in the figures.

To test whether serum CD5L correlates to alternative indicators of TH action, the concentrations of CD5L were compared with total serum Se, total serum Cu, total serum SELENOP, and total serum CP. In addition, the products of serum Se and Cu along with the product of serum SELENOP and CP were compared. The results indicate a significant positive association of CD5L with serum Se, SELENOP, and Cu, but not with CP or the product of CP and SELENOP in this cohort of pregnant women (Figure 5). Notably, strongest correlation was observed for CD5L and the product of serum Cu and Se concentration (Figure 5C).

## 4 DISCUSSION

In this study, we describe the generation of a novel sandwich ELISA for human CD5L, its technical characterization, and a first application in three clinical cohort studies to test the potential suitability of serum CD5L as additional biomarker of TH status. The results indicate that serum CD5L is not strongly affected by the time of blood drawing, that is, does not follow a stringent circadian rhythm, which is an advantage for being considered as a potentially useful serum biomarker. Moreover, it seems to be unaffected by liver disease as evidenced by the lack of correlation to disease severity in patients with liver cirrhosis, despite its mainly hepatic origin. And importantly, serum CD5L proved to be positively related to fT3 concentrations in serum of pregnant women, much in agreement with our hypothesis and with prior research on its regulation by TH in liver.^15^

Interestingly, serum CD5L was not significantly associated with serum fT4 or TSH values supporting its peripheral origin, not subject to a control of THRB signaling only, but rather regulated by the activity of both receptor isoforms, that is, THRA and THRB.^15^ In view of our primary study aim, that is, the identification of a peripheral biomarker of TH action, the correlation to fT3 but not to T4 or TSH accords with recent results highlighting its specific importance from large‐scale observational studies. In the general population, fT3 but not T4 or TSH, proved as a meaningful biomarker of longevity and social status,^26^ while in pregnancy, fT3 was specifically associated with preeclampsia and gestational hypertension risk.^27^ The relationship of CD5L with fT3 in serum of pregnant women was further underlined by its positive correlation to serum concentrations of Se, SELENOP, and Cu, known to positively reflect liver TH status.^13^, ^14^ Notably, serum Se and SELENOP have been identified as THRA‐dependent TH targets, whereas serum Cu rather reflects THRB‐dependent TH action in liver. While the positive association of TH with serum Se and SELENOP was replicated in the samples from pregnant women, a correlation of TH to the Cu transport protein CP was not observed in pregnancy, despite the prior findings in rodents and humans.^14^ The positive effects of pregnancy‐associated estradiol increase may account for this apparent difference.^28^

According to the theory of CD5L as integrating hepatic TH activity, the product of both trace elements as readout of an activation of both TH receptors showed the strongest correlation to serum CD5L. It was surprising to see these tight correlations of CD5L to the trace elements, as both serum Se and Cu are regulated by inflammation, notably in opposite directions, with serum Se representing a negative and Cu a positive acute phase reactant. Hence, the observed correlation of CD5L to the Cu times Se product indicates a strong and dominant effect of fT3 on these biomarkers, overriding any potential inflammatory influences during pregnancy which would diminish the collinear correlation of Se and Cu with CD5L. Collectively, the data support the concept of CD5L as an additional biomarker of peripheral TH action, in particular in pregnancy, largely independent from central THRB‐dependent feedback on the routine TH biomarkers fT4 and TSH. The observed correlation constitutes a particularly promising finding, as fT3 appears to be specifically related to human physiology, aging, and longevity, displaying only little relation to TSH or fT4 concentrations in healthy subjects.^26^

The consistency of the associations of serum CD5L with fT3 in pregnancy was unexpected in view of the many endogenous and pathophysiological pathways potentially affecting CD5L concentrations in blood. Previous studies have identified serum CD5L as a protein derived mainly from liver macrophages.^16^ It was initially described as apoptosis inhibitor expressed by macrophages and has since been characterized to be highly responsive to cytokine signaling.^16^, ^29^ In lung cancer patients, CD5L is detected in extracellular vesicles of liquid biopsies and positively correlated to tissue concentrations and disease severity.^30^ In chronic kidney disease, serum CD5L is associated with cardiovascular events and all‐cause mortality.^31^ In patients with hepatocellular carcinoma, CD5L concentrations correlate to relapse‐free and overall survival.^32^ Reduced serum CD5L has also been described in acute‐on‐chronic liver failure,^33^ as a biomarker for latent steatohepatitis and cancer risk,^34^ and as a component of circulatory immunoglobulin M (IgM).^35^ Therapeutic antibodies to CD5L are described as potential tumor drugs displaying promising chemotherapeutic effects.^36^

While all these studies highlight different and relevant physiological regulators of CD5L, our results suggest a dominant association of serum CD5L with TH status during pregnancy, despite the strong and dynamic adaptations of the endocrine and immune system, including the circulating steroid hormone and cytokine concentrations, along gestation.^37^ In order to better understand the balance of the different regulators of CD5L expression and secretion and to identify even stronger modulators than TH, larger studies with a more diverse set of subjects will be needed. This line of further research is strongly supported by the reliability of the new assay developed, the favorable characteristics of CD5L as stable serum protein, and the consistent findings concerning the fT3‐dependence as observed in our prior study with hypo‐ and hyperthyroid patients,^15^ and in the current analysis in pregnancy. Yet, in order to establish its potential diagnostic value during pregnancy, larger studies ideally assessing samples taken at different time points of women with and without thyroid dysfunction will be needed.

Among the strengths of the study are the high quality of the newly generated mAbs and quantification assays, along with the stability of CD5L in serum that allows affordable high throughput analyses of large cohort studies, even when samples had been frozen and thawed before. The apparent stability toward circadian rhythms renders CD5L as a convenient biomarker with little requirements toward the timing of sample preparation.

Among the notable limitations are the single time point analyzed during pregnancy, a lack of data on other potential biomarkers of peripheral TH action, such as sex‐hormone binding globulin (SHBG), ferritin, fibroblast growth factor‐21 (FGF‐21), or others,^12^ and no information on child growth and performance as a definitive endpoint of euthyroidism or altered TH status during pregnancy. Moreover, trimester‐specific data on the variation of CD5L during pregnancy are not yet at hand, it is unknown whether it qualifies as biomarker in women under T4 treatment, and in particular whether it provides diagnostic information in pregnant women with thyroid or hepatic disease. To this end, additional and sufficiently powered analytical studies are needed to specify the indications where the assessment of CD5L as biomarker of TH action in addition to TSH and fT4 is of diagnostic value, versus those conditions where it does not add information to the clinical workup. Moreover, our notion on little influence of age and sex on circulating CD5L concentrations has been derived from patients with liver disease only, and is in need of replication in other groups of healthy and diseased male and female subjects spanning a wider age range. Nevertheless, the newly generated assay and the data from this study support the consideration of CD5L as a potentially meaningful and suitable biomarker of integrated TH activity in liver, thereby nicely complementing TH status assessment with an additional readout for peripheral TH activity.

## Supporting information


**TABLE S1:** Circadian variation in serum CD5L concentrations.

## Data Availability

The raw data supporting the conclusions of this article will be made available by the authors upon reasonable request to the corresponding author.

## References

[1] Ortiga‐Carvalho TM , Chiamolera MI , Pazos‐Moura CC , Wondisford FE . Hypothalamus‐pituitary‐thyroid Axis. Compr Physiol. 2016;6(3):1387–1428. 27347897 10.1002/cphy.c150027

[2] Vanhaelst L , Van Cauter E , Degaute JP , Golstein J . Circadian variations of serum thyrotropin levels in man. J Clin Endocrinol Metab. 1972;35(3):479–482. 5051370 10.1210/jcem-35-3-479

[3] Hoermann R , Midgley JEM , Larisch R , Dietrich JW . Recent advances in thyroid hormone regulation: toward a new paradigm for optimal diagnosis and treatment. Front Endocrinol (Lausanne). 2017;8:364. 29375474 10.3389/fendo.2017.00364 PMC5763098

[4] Bianco AC . We all know we need them, we Hope they are coming, but when? Thyroid. 2020;30(6):791–793. 32228170 10.1089/thy.2020.0250 PMC7307695

[5] Moran C , Chatterjee K . Resistance to thyroid hormone due to defective thyroid receptor alpha. Best Pract Res Clin Endocrinol Metab. 2015;29(4):647–657. 26303090 10.1016/j.beem.2015.07.007 PMC4559105

[6] Dumitrescu AM , Liao XH , Abdullah MS , Lado‐Abeal J , Majed FA , Moeller LC , et al. Mutations in SECISBP2 result in abnormal thyroid hormone metabolism. Nat Genet. 2005;37(11):1247–1252. 16228000 10.1038/ng1654

[7] Friesema EC , Grueters A , Biebermann H , Krude H , von Moers A , Reeser M , et al. Association between mutations in a thyroid hormone transporter and severe X‐linked psychomotor retardation. Lancet. 2004;364(9443):1435–1437. 15488219 10.1016/S0140-6736(04)17226-7

[8] Korevaar TIM , Medici M , Visser TJ , Peeters RP . Thyroid disease in pregnancy: new insights in diagnosis and clinical management. Nat Rev Endocrinol. 2017;13(10):610–622. 28776582 10.1038/nrendo.2017.93

[9] Lee SY , Pearce EN . Assessment and treatment of thyroid disorders in pregnancy and the postpartum period. Nat Rev Endocrinol. 2022;18(3):158–171. 34983968 10.1038/s41574-021-00604-z PMC9020832

[10] Jansen HI , van Herwaarden AE , Huijgen HJ , Painter RC , Hillebrand JJ , Boelen A , et al. Pregnancy disrupts the accuracy of automated fT4 immunoassays. Eur Thyroid J. 2022;11(6):e220145. 36219545 10.1530/ETJ-22-0145 PMC9641786

[11] Ollero MD , Toni M , Pineda JJ , Martínez JP , Espada M , Anda E . Thyroid function reference values in healthy iodine‐sufficient pregnant women and influence of thyroid nodules on thyrotropin and free thyroxine values. Thyroid. 2019;29(3):421–429. 30693851 10.1089/thy.2018.0324

[12] Jansen HI , Bruinstroop E , Heijboer AC , Boelen A . Biomarkers indicating tissue thyroid hormone status: ready to be implemented yet? J Endocrinol. 2022;253(2):R21–R45. 35256536 10.1530/JOE-21-0364

[13] Mittag J , Behrends T , Hoefig CS , Vennstrom B , Schomburg L . Thyroid hormones regulate selenoprotein expression and selenium status in mice. PLoS One. 2010;5(9):e12931. 20877559 10.1371/journal.pone.0012931 PMC2943913

[14] Mittag J , Behrends T , Nordstrom K , Anselmo J , Vennstrom B , Schomburg L . Serum copper as a novel biomarker for resistance to thyroid hormone. Biochem J. 2012;443(1):103–109. 22220593 10.1042/BJ20111817

[15] Nock S , Johann K , Harder L , Wirth EK , Renko K , Hoefig CS , et al. CD5L constitutes a novel biomarker for integrated hepatic thyroid hormone action. Thyroid. 2020;30:908–923. 32183611 10.1089/thy.2019.0635

[16] Sanjurjo L , Aran G , Roher N , Valledor AF , Sarrias MR . AIM/CD5L: a key protein in the control of immune homeostasis and inflammatory disease. J Leukoc Biol. 2015;98(2):173–184. 26048980 10.1189/jlb.3RU0215-074R

[17] Oliveira L , Silva MC , Gomes AP , Santos RF , Cardoso MS , Nóvoa A , et al. CD5L as a promising biological therapeutic for treating sepsis. Nat Commun. 2024;15(1):4119. 38750020 10.1038/s41467-024-48360-8 PMC11096381

[18] Arai S , Kitada K , Yamazaki T , Takai R , Zhang X , Tsugawa Y , et al. Apoptosis inhibitor of macrophage protein enhances intraluminal debris clearance and ameliorates acute kidney injury in mice. Nat Med. 2016;22(2):183–193. 26726878 10.1038/nm.4012

[19] Kurokawa J , Arai S , Nakashima K , Nagano H , Nishijima A , Miyata K , et al. Macrophage‐derived AIM is endocytosed into adipocytes and decreases lipid droplets via inhibition of fatty acid synthase activity. Cell Metab. 2010;11(6):479–492. 20519120 10.1016/j.cmet.2010.04.013

[20] Wilms B , Leineweber EM , Mölle M , Chamorro R , Pommerenke C , Salinas‐Riester G , et al. Sleep loss disrupts morning‐to‐evening differences in human white adipose tissue transcriptome. J Clin Endocrinol Metab. 2019;104(5):1687–1696. 30535338 10.1210/jc.2018-01663

[21] Filipowicz D , Szczepanek‐Parulska E , Klobus M , Szymanowski K , Chillon TS , Asaad S , et al. Selenium status and supplementation effects in pregnancy‐a study on mother‐child pairs from a single‐center cohort. Nutrients. 2022;14(15):3082. 35956267 10.3390/nu14153082 PMC9370234

[22] Filipowicz D , Szczepanek‐Parulska E , Mikulska‐Sauermann AA , Karaźniewicz‐Łada M , Główka FK , Szymanowski K , et al. Iodine deficiency and real‐life supplementation ineffectiveness in polish pregnant women and its impact on thyroid metabolism. Front Endocrinol (Lausanne). 2023;14:1068418. 37396162 10.3389/fendo.2023.1068418 PMC10313195

[23] Hybsier S , Schulz T , Wu Z , Demuth I , Minich WB , Renko K , et al. Sex‐specific and inter‐individual differences in biomarkers of selenium status identified by a calibrated ELISA for selenoprotein P. Redox Biol. 2017;11:403–414. 28064116 10.1016/j.redox.2016.12.025 PMC5220167

[24] Hackler J , Wisniewska M , Greifenstein‐Wiehe L , Minich WB , Cremer M , Bührer C , et al. Copper and selenium status as biomarkers of neonatal infections. J Trace Elem Med Biol. 2020;58:126437. 31778962 10.1016/j.jtemb.2019.126437

[25] Kuhn EC , Slagman A , Kuhn‐Heid ECD , Seelig J , Schwiebert C , Minich WB , et al. Circulating levels of selenium‐binding protein 1 (SELENBP1) are associated with risk for major adverse cardiac events and death. J Trace Elem Med Biol. 2019;52:247–253. 30732890 10.1016/j.jtemb.2019.01.005

[26] Lawton RI , Sabatini BL , Hochbaum DR . Longevity, demographic characteristics, and socio‐economic status are linked to triiodothyronine levels in the general population. Proc Natl Acad Sci U S A. 2024;121(2):e2308652121. 38175866 10.1073/pnas.2308652121 PMC10786306

[27] Derakhshan A , Männistö T , Chen L , Osinga JAJ , Ashoor G , Lu X , et al. Association of gestational free and total triiodothyronine with gestational hypertension, preeclampsia, preterm birth, and birth weight: an individual participant data meta‐analysis. J Clin Endocrinol Metab. 2024;109(3):e1290–e1298. 37878891 10.1210/clinem/dgad631 PMC10876397

[28] Sparre LS , Brundin J , Carlström A , von Schoultz B , Carlström K . Serum levels of estrogens and of five ‘steroid sensitive’ proteins in early normal pregnancy. Acta Endocrinol. 1988;118(2):239–244. 10.1530/acta.0.1180239 2455429

[29] Barcena C , Aran G , Perea L , Sanjurjo L , Tellez E , Oncins A , et al. CD5L is a pleiotropic player in liver fibrosis controlling damage, fibrosis and immune cell content. EBioMedicine. 2019;43:513–524. 31076347 10.1016/j.ebiom.2019.04.052 PMC6558273

[30] Choi ES , Faruque HA , Kim JH , Kim KJ , Choi JE , Kim BA , et al. CD5L as an extracellular vesicle‐derived biomarker for liquid biopsy of lung cancer. Diagnostics (Basel). 2021;11(4):620. 33808296 10.3390/diagnostics11040620 PMC8067192

[31] Castelblanco E , Sarrias MR , Betriu À , Soldevila B , Barranco‐Altirriba M , Franch‐Nadal J , et al. Circulating CD5L is associated with cardiovascular events and all‐cause mortality in individuals with chronic kidney disease. Aging (Albany NY). 2021;13(19):22690–22709. 34629330 10.18632/aging.203615 PMC8544330

[32] Luo Y , Huang X , Zhan J , Zhang S . Role of CD5L and SRD5A2 as prognostic biomarkers for hepatocellular carcinoma. Int J Gen Med. 2021;14:9247–9260. 34880664 10.2147/IJGM.S337769 PMC8646114

[33] Sánchez‐Rodríguez MB , Téllez É , Casulleras M , Borràs FE , Arroyo V , Clària J , et al. Reduced plasma extracellular vesicle CD5L content in patients with acute‐on‐chronic liver failure: interplay with specialized pro‐resolving lipid mediators. Front Immunol. 2022;13:842996. 35330909 10.3389/fimmu.2022.842996 PMC8940329

[34] Okanoue T , Yamaguchi K , Shima T , Mitsumoto Y , Mizuno M , Katayama T , et al. Serum levels of immunoglobulin M‐free inhibitors of macrophage/CD5L as a predictive and early diagnostic marker for nonalcoholic steatohepatitis‐associated hepatocellular carcinoma. Hepatol Res. 2022;52(12):998–1008. 35939571 10.1111/hepr.13826

[35] Oskam N , den Boer MA , Lukassen MV , Ooijevaar‐de Heer P , Veth TS , van Mierlo G , et al. CD5L is a canonical component of circulatory IgM. Proc Natl Acad Sci U S A. 2023;120(50):e2311265120. 38055740 10.1073/pnas.2311265120 PMC10723121

[36] Sanchez‐Moral L , Paul T , Martori C , Font‐Díaz J , Sanjurjo L , Aran G , et al. Macrophage CD5L is a target for cancer immunotherapy. EBioMedicine. 2023;91:104555. 37054630 10.1016/j.ebiom.2023.104555 PMC10139961

[37] Piccinni MP , Raghupathy R , Saito S , Szekeres‐Bartho J . Cytokines, hormones and cellular regulatory mechanisms favoring successful reproduction. Front Immunol. 2021;12:717808. 34394125 10.3389/fimmu.2021.717808 PMC8355694

