# Supplementary material for: Serum CD5L as potential biomarker of thyroid hormone status during pregnancy
Source: Biofactors. 2024 Sep 30;51(1):e2123. doi: 10.1002/biof.2123 (PMC11681307; doi:10.1002/biof.2123)
Supplement: Supplementary file 1 — TABLE S1: Circadian variation in serum CD5L concentrations. [file BIOF-51-0-s001.docx]

**Supplementary Table**

Circadian variation in serum CD5L concentrations

| Subject | n | median | mean | SD | CV (%) |
| --- | --- | --- | --- | --- | --- |
| 1 | 16 | 2.22 | 2.27 | 0.34 | 14.91 |
| 2 | 16 | 3.03 | 3.02 | 0.11 | 3.53 |
| 3 | 13 | 2.66 | 2.68 | 0.25 | 9.30 |
| 4 | 16 | 2.25 | 2.28 | 0.17 | 7.54 |
| 5 | 16 | 2.42 | 2.42 | 0.22 | 8.88 |
| 6 | 16 | 3.07 | 3.10 | 0.21 | 6.85 |
| 7 | 16 | 1.91 | 1.89 | 0.17 | 9.21 |
| 8 | 15 | 1.86 | 1.86 | 0.14 | 7.66 |
| 9 | 16 | 2.55 | 2.54 | 0.13 | 5.06 |
| 10 | 16 | 2.58 | 2.60 | 0.19 | 7.51 |
